# Genetic Differences in Reactivity to the Environment Impact Psychotic-Like and Affective Reactivity in Daily Life

**DOI:** 10.1093/schbul/sbad162

**Published:** 2025-03-04

**Authors:** Neus Barrantes-Vidal, Pilar Torrecilla, Patricia Mas-Bermejo, Sergi Papiol, Marian J Bakermans-Kranenburg, Araceli Rosa, Thomas R Kwapil

**Affiliations:** Departament de Psicologia Clínica i de la Salut, Universitat Autònoma de Barcelona, Barcelona, Spain; CIBER de Salud Mental, Instituto de Salud Carlos III, Madrid, Spain; Departament de Psicologia Clínica i de la Salut, Universitat Autònoma de Barcelona, Barcelona, Spain; Secció de Zoologia i Antropologia Biològica, Departament de Biologia Evolutiva, Ecologia i Ciències Ambientals, Universitat de Barcelona, Barcelona, Spain; Institut de Biomedicina de la UB (IBUB), Barcelona, Spain; CIBER de Salud Mental, Instituto de Salud Carlos III, Madrid, Spain; Institute of Psychiatric Phenomics and Genomics (IPPG), University Hospital, LMU Munich, Munich 80336, Germany; Max Planck Institute of Psychiatry, Munich 80804, Germany; ISPA, University Institute of Psychological, Social and Life Sciences, Lisbon, Portugal; Department of Psychology, Personality, Social and Developmental Psychology, Stockholm University, Stockholm, Sweden; CIBER de Salud Mental, Instituto de Salud Carlos III, Madrid, Spain; Secció de Zoologia i Antropologia Biològica, Departament de Biologia Evolutiva, Ecologia i Ciències Ambientals, Universitat de Barcelona, Barcelona, Spain; Institut de Biomedicina de la UB (IBUB), Barcelona, Spain; Department of Psychology, University of Illinois at Urbana-Champaign, Champaign, IL, USA

**Keywords:** schizotypy, psychosis, gene–environment interaction, experience sampling methodology, differential susceptibility, Polygenic Risk Score

## Abstract

**Background and Hypothesis:**

Consistent with diathesis-stress models, psychosis research has focused on genetic moderation of *adverse* environmental exposures. In contrast, the Differential Susceptibility (DS) model suggests that the same genetic variants that increase risk-inducing effects of adverse experiences also enhance beneficial effects from positive experiences. This study examined whether individuals with high genetic susceptibility to the environment showed differential psychotic-like and affective reactivity in response to positive and negative events in daily life.

**Study Design:**

Experience sampling methodology assessed context (positive and stressful) and momentary levels of paranoia, psychotic-like experiences (PLE), and positive (PA) and negative affect (NA) in 217 non-clinical adults oversampled for schizotypy. Linear mixed models examined whether Polygenic Risk Scores of Environmental Sensitivity (PRS-ES) moderated the impact of current context on subsequent experiences.

**Study Results:**

PRS-ES moderated positive, but not stressful, context on subsequent levels of momentary paranoia, NA, and PA, but not PLE. Genetic and environmental (G × E) interactions indicated diathesis-stress at lower thresholds of PRS-ES, but a DS model at the highest threshold of the PRS-ES. Participants with elevated PRS-ES showed increased paranoia and NA and decreased PA in subsequent assessments when reporting low levels of positive situations, but also decreased paranoia and NA and increased PA when rating contexts as positive.

**Conclusions:**

Findings support the influence of genetic sensitivity to the environment on psychotic-like and affective reactivity in daily life, particularly in response to positive contexts. This highlights the transdiagnostic protective role of positive experiences and informs ecological momentary interventions.

## Introduction

The extended psychosis-proneness phenotype, referred to as schizotypy, ranges in expression from minimal dysfunction (eg, psychotic-like experiences; PLE) to full-blown psychosis (eg, schizophrenia).^1–4^ Evidence suggests that vulnerability factors to the psychosis-spectrum phenotype are shared across a broad range of psychotic and non-psychotic phenotypes^5^ at both genetic^6–8^ and environmental^9–13^ levels.

The study of the interplay between genetic and environmental (G × E) etiological factors in psychosis has been mainly guided by the traditional diathesis-stress framework^14,15^ that posits that individuals carrying genetic risk variants are more vulnerable to the effects of negative environments and more prone to develop psychopathology. This framework has neglected the role of positive experiences. However, emerging evidence supports the positive impact of supportive experiences attenuating psychosis-spectrum expression.^16–18^ In this regard, novel thinking based on evolutionary theory suggests that individuals may differ in their susceptibility to the environment across a broad range of exposures (not just negative) and, therefore, moderation effects should also be expected for positive experiences. The Differential Susceptibility (DS) model proposes that the same genetic variants and biological or temperamental traits that increase the negative effects of adverse experiences also enhance the likelihood of benefiting from positive experiences and, thus, that some individuals may be more susceptible, plastic or malleable to the environment.^19–21^ Of note, the DS model integrates the classic diathesis-stress perspective, focused on the negative side of environment, as well as its mirror image, vantage sensitivity, a model exclusively focused on the beneficial effects of positive environments. However, the latter seems not to be as strongly supported as DS by theoretical or evolutionary background.^22^

Research has supported the DS model for several phenotypes,^23–25^ but only one previous study has tested the validity of DS for schizotypy and PLE.^26^ This is possibly related to the fact that psychosis research is only starting to attend to effects of positive environmental exposures.^16–18^ Barrantes-Vidal et al^^26^^ showed that non-clinical young adults who were highly genetically sensitive to the environment, compared to those with low genetic sensitivity, displayed increased levels of positive schizotypy and PLEs, depression and anxiety if they reported high levels of childhood adversity but, at the same time, fewer symptoms if they reported low or no levels of adversity.

DS research has mostly focused on long-term developmental changes, that is, how early-life experiences affect an individual’s developmental trajectory. Another level of analysis that has received little empirical attention involves short-term influences, such as immediate effects of stimuli on behavior. This approach has been referred to as differential reactivity.^27^ Differential reactivity involves more transient behavioral changes, which have also been referred to as “activational”^28^ or “contextual”^29^ plasticity. Research on differential reactivity has mostly relied on experimental manipulations of the environment,^22,30–32^ but it can also be explored in relation to immediate normally occurring stimuli or daily life events.^27^ In this regard, ambulatory assessment techniques such as experience sampling methodology (ESM) may be optimal for the examination of dynamic, within-person environmental reactivity.

ESM is a within-day self-assessment technique used to capture cognition, affect, symptoms, and contextual factors.^33^ Repeatedly assessing participants’ experiences in real-time and in the real-world minimizes retrospective bias and enhances ecological validity.^34,35^ ESM has successfully been used to examine psychotic and affective reactivity to stress across clinical^36–38^ and nonclinical^39–43^ samples. More so, stress-related genes have shown to moderate such psychotic reactivity in the flow of daily life.^44–46^ However, to the best of our knowledge only one study has examined genetic differential reactivity to both positive and negative daily events.^47^ That study tested whether carriers of the short allele (S) of the 5-HTTLPR variant, one of the variants used as a proxy genetic indicator of plasticity and DS effects,^23,48^ moderated the reactivity to both uplifts and stressors. Contrary to expectations, carriers of the S allele showed less reactivity than the homozygous carriers of the long allele (L/L), which was interpreted in the context of the unsuccessful replicability efforts of candidate-gene studies (and particularly, of 5-HTTLPR). In this regard, modern polygenic approaches have been suggested to greatly improve our understanding of the role of G × E in psychopathology,^49,50^ and more so in combination with real-time measurement of individuals’ context and mental state.^51,52^

The present study examined differential reactivity in daily life by testing, for the first time, whether a Polygenic Risk Score of Environmental Sensitivity (PRS-ES)^53^ moderated the association of momentary appraisals of the current context (both positive and stressful) with subsequent momentary reports of subclinical psychotic experiences (positive, paranoid, and negative dimensions) as well as affective (positive and negative affect) manifestations in a DS manner.

It was expected that highly genetically sensitive individuals who rated their current context as stressful or as not positive would show subsequent greater levels of PLE, paranoia, and negative affect, as well as lower positive affect, as compared to less genetically sensitive individuals. Moreover, genetically highly sensitive individuals were expected to show lower levels of subsequent symptoms and more positive affect when they rated their current context as minimally stressful or highly positive. Given that the positive and paranoid, but not the negative, dimensions of schizotypy, and clinical psychosis have been associated with increased stress-induced psychotic reactivity,^39–44,54–56^ and that the negative dimension is characterized by diminished motivation and low openness to experience,^57,58^ we hypothesized that this genetically moderated reactivity to context would not be observed for momentary negative PLE. Furthermore, findings from the previous study examining DS in relation to schizotypy and PLE using retrospective measures did not find an association with negative schizotypy.^26^

## Methods

### Participants

The sample of the present study consisted of 217 (mean age = 21.92, SD = 2.78; 75% female) non-clinical participants belonging to the Barcelona Longitudinal Investigation of Schizotypy Study (BLISS).^39,57^ At T1, a large pool of 547 unselected college students and 261 technical school students were initially screened with self-report questionnaires. At T2, a subsample 214 college and 39 technical school students oversampled for schizotypy scores was selected to conduct in depth examinations comprising a wide range of interview, questionnaire, and ESM measurements. Usable ESM data were available for a total of 206 college and 36 technical school students (see details in Racioppi et al^59^). At T2 genetic data was also collected. After genetic quality control, the sample with usable genetic and ESM data comprised a total of 217 non-clinical young adults (197 from college and 20 from technical schools).

### Materials and Procedure

#### Calculation of PRS

DNA was extracted from saliva or cotton swabs samples and genotyped using the Illumina Infinium Global Screening Array-24 v2.0 (GSA) BeadChip at the “Centro Nacional de Genotipado” (CEGEN-PRB3-ISCIII; CNIO-Madrid). The details about the genotyping, quality control, and imputation procedures can be found in Supplementary Materials. Polygenic Risk Scores (PRS) were computed based on Genome Wide Association Study (GWAS) of reference by summing the number of risk alleles that participants carried multiplied by their effect sizes. We calculated a PRS-ES based on Keers et al^53^ GWAS who conducted a monozygotic twin study that captured genetic variants associated to differences in emotional (internalizing) symptoms within twins’ pairs. This design allowed to attribute symptom differences to genetic susceptibility to non-shared environmental factors and therefore, capture environmental sensitivity.^53^

We applied the classical Clumping + Thresholding (C + T) method with PLINK v1. 9 (www.cog-genomics.org/plink/1.9/;).^60^ Independent variants were selected by clumping (*r*^2^ < .1 within a 1000 kb window) using the 1000 Genomes Project phase 3^61^ as a European linkage disequilibrium (LD) reference panel and 93 494 SNPs survived clumping. We obtained scores with *P*-value thresholds of .001, .01, .05 and .1, based on previous G × E evidence using PRS-ES.^26,^^62^ The PRS-ES was computed based on 369 SNPs for *P* < .001; 2819 SNPs for *P* < .01; 11 244 SNPs for *P* < .05; and 19 895 SNPs for *P* < .10.

#### Experience Sampling Methodology

ESM data were collected on personal digital assistants (PDAs) that signaled participants randomly eight times a day (between 10 a.m. and 10 p.m.) for one week to complete short questionnaires enquiring about a variety of daily life experiences. Participants had 5 min to initiate responding to the questionnaire following the signal, after this period or completion of the questionnaire, the PDA was shut down until next signal. Consecutive survey notifications could range from 10 to 170 min apart. The complete list of ESM items can be found in Barrantes-Vidal et al.^39^ All items were rated on 7-point scales from “not at all” to “very much.” In the present study, reports of momentary paranoia, PLE, diminished thoughts/emotions (as a proxy of negative psychotic symptoms), and negative (NA) and positive (PA) affect were employed as outcome measures. Two separate items were used to rate how positive and how stressful the current situation was, as indicators of current context. Details on indices, items, and reliabilities are presented in table 1.

**Table 1.** ESM Items Used in This Study

Domain	ESM Items	Reliability
Outcomes		
Paranoia index	Computed as the mean of 2 items: Right now, I feel suspicious Right now, I feel mistreated	Between α = .75 Within α = .46
Psychotic-like experiences index (PLE)	Computed as the mean of 8 items: Right now, I fear losing control Right now, I feel weird Right now, I have difficulty controlling my thoughts Right now, my thoughts are strange or unusual Right now, my sight or hearing seem strange or unusual Since the last beep, I have heard or seen things others could not Right now, I feel that someone or something is controlling my thoughts or actions Right now, familiar things seem strange and unusual	Between α = .79 Within α = .49
Negative-like symptoms	Right now, I have no thoughts or emotions	—
Negative affect index (NA)	Computed as the mean of 4 items: Right now, I feel sad Right now, I feel anxious (nervous) Right now, I feel angry Right now, I feel guilty or ashamed	Between α = .81 Within α = .58
Positive affect index (PA)	Computed as the mean of 2 items: Right now, I feel happy Right now, I feel relaxed	Between α = .81 Within α = .46
Contextual predictors		
Stressful situation	My current situation is stressful	—
Positive situation	My current situation is positive	—

### Statistical Analysis

ESM data have a hierarchical structure in which ESM ratings (level 1 data) are nested within participants (level 2 data). As the standard approach for the analysis of ESM data, linear mixed models were used to control for within-subject clustering of multiple observations using Version 3.6.3 of the LEGIT package^63^ in R.^64^ The present study examined (1) the time-lagged association between the current ratings of situations as (a) positive and (b) stressful (time *t*) on levels of criteria at the subsequent ESM assessment (time *t* + 1), and (2) the cross-level interaction between level 2 genetic data (PRS-ES) and appraisals of the situation at time *t* on criteria at time *t* + 1. Time-lagged analyses were limited to examining within-day associations. Moreover, cases with missing data on relevant variables at time *t* or time *t* + 1 were excluded from each analysis. Interactions yielding significant effects (*P* values < .05) were examined based on the competitive-confirmatory approach^65,66^ to determine whether the G × E interaction fitted a DS model. As detailed elsewhere,^26^ the LEGIT package envisions weak and strong versions of three G × E models (DS, diathesis-stress and vantage sensitivity), and the model showing lowest Akaike Information Criterion (AIC) represents the best fit. To classify an interaction within the DS model, the 95% interval of its estimated crossover point needs to be within observable bounds of the environmental score.

All analyses included the first two ancestry-informative principal components from the Multidimensional Scaling analysis, Principal Component 1 (PC1) and 2 (PC2), as covariates in the mixed models examined and were trimmed from the competitive-confirmatory test phase if they were nonsignificant. *T*-tests for independent samples did not reveal any significant difference in PRS or ancestry covariates between the two samples used (ie, college and technical school students). We used False Discovery Rate (FDR)^67^ to correct for multiple testing across thresholds of PRS-ES for each of the outcome variables.

## Results

Participants completed an average of 40.7 questionnaires (SD = 9.1). Table 2 shows the descriptive statistics and the Pearson correlations of the study variables. PRS-ES (thresholds *P* < .001; .05; .10) showed small effect size correlations with positive appraisals of the current situation and PA (thresholds *P* < .001; .05) with values ranging from *r* = −.10 to *r* = −.18. The PRS-ES (threshold *P* < .10) also showed some association with PLE (*r* = .15). Following Belsky et al’s^19^ stepwise testing approach for DS, the potential susceptibility factor (here, PRS-ES) should not be associated with the environmental predictor or the outcome. However, the examination of the confidence intervals (see Supplementary table 1) indicates that all the correlation values fall within the other correlations’ confidence intervals—indicating that the six correlation values do not significantly differ (and should essentially be considered equivalent). Although the correlation values attained statistical significance, these are small effects indicating only 1%–3% of shared variance, and minimal collinearity between the ESM items and the PRS-ES. Thus, such small effects and minimal collinearity should not preclude the examination of the interaction of the PRS-ES and ESM scores from a DS perspective.

**Table 2.** Descriptive Statistics and Pearson Correlations for the Study Variables (*n* = 217)

	Descriptive Statistics		Pearson Correlations (*r*)										
	*M* (SD)	Range	1	2	3	4	5	6	7	8	9	10	11
1. PRS-ES (*P* < .001)	0.88 (1.04)	−2.03 to 3.89	—	.39***	.40***	.37***	−.00	−.14*	−.04	−.00	−.01	.05	−.15*
2. PRS-ES (*P* < .01)	4.13 (2.25)	−1.55 to 10.76		—	.67***	.61***	−.02	−.10	.07	.09	−.05	.04	−.05
3. PRS-ES (*P* < .05)	6.70 (4.73)	−5.39 to 20.08			—	.88***	.03	−.18**	.12	.11	.07	.11	−.14*
4. PRS-ES (*P* < .10)	9.01 (5.64)	−3.31 to 25.15				—	−.02	−.17*	.10	.15*	.08	.08	−.08
5. ESM Stressful situation	2.15 (1.06)	1–6.31					—	−.39***	.36***	.39***	.01	.62***	−.53***
6. ESM positive situation	5.35 (0.96)	1.91–7						—	−.36***	−.29***	−.10	−.53***	.81***
7. ESM Paranoia	1.21 (0.35)	1–3.25							—	.70***	.18***	.76***	−.34***
8. ESM PLE	1.12 (0.18)	1–2.63								—	.24***	.58***	−.28***
9. ESM negative symptoms	1.35 (0.69)	1–4.90									—	.09	−.01
10. ESM negative affect	1.51 (0.47)	1–3.92										—	−.56***
11. ESM positive affect	4.70 (0.79)	2.57–6.47											—

*Note*: Mean ESM scores for each participant are reported. *r* > .30 are medium effect sizes and *r* > .50 are large effect sizes. ESM, experience sampling methodology; PRS-ES, Polygenic Risk Score of Environmental Sensitivity.

*P < .05, **P < .01, ***P < .001.

**Fig. 1.**
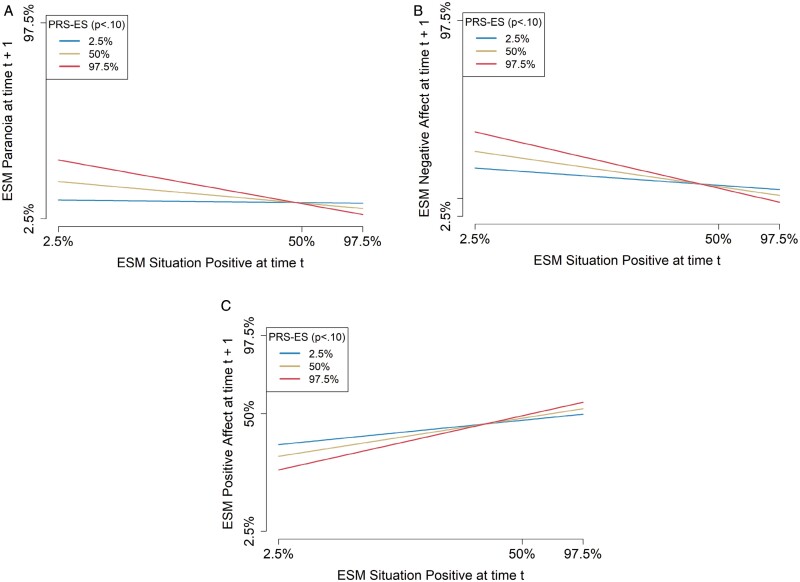
Graphic representation of the significant interactions between PRS-ES (*P* < .10) and ratings of positive situation on subsequent momentary (a) paranoia, (b) negative affect, and (c) positive affect. ESM, experience sampling methodology; PRS-ES, Polygenic Risk Score.

As shown in table 3, ratings of the current situation as positive were associated with subsequent levels of momentary paranoia, PLE, negative-like symptoms, NA, and PA. After adjusting for multiple testing, PRS-ES moderated the association between positive situation and subsequent momentary paranoia (thresholds *P* < .01; .05; .10), NA (thresholds *P* < .01; .05; .10) and PA (threshold *P* < .01; .05; .10). For the interactions on subsequent levels of paranoia, the competitive-confirmatory analyses revealed evidence for strong DS for the greater polygenic thresholds (thresholds *P* < .05 and .10) and diathesis-stress for the less polygenic threshold *P* < .01. That is, participants with higher (especially more polygenic; thresholds *P* < .05 and .10) PRS-ES scores showed increased levels of subsequent paranoia when in low positive contexts, but also less paranoia when the context was positive (figure 1a), whereas those with less polygenic PRS-ES (threshold *P* < .01) were only affected by low, not high, positive contexts, which increased their subsequent paranoia. In other words, only participants with more polygenic PRS-ES (thresholds *P* < .05 and .10) showed paranoid reactivity in the form of *both* increases and decreases in momentary paranoia in response to the context (rated from low to high positive, respectively). Similarly, interaction effects between PRS-ES and context on subsequent levels of negative as well as positive affect were classified as fitting a weak model of DS for the most polygenic threshold (*P* < .10), but a weak diathesis-stress pattern for lower thresholds (*P* < .01 and .05). Thus, participants with a more polygenic PRS-ES (threshold *P* < .10) showed reduced PA and increased NA if the situation was not positive, but increased PA and reduced NA if the situation was highly positive (figure 1b and c). However, at lower thresholds of PRS-ES, participants were only affected by low positive contexts, showing subsequent reduced PA and increased NA.

**Table 3.** Effects of PRS of Environmental Sensitivity (*P* < .001, .01, .05, .10), Positive Situation and Their Interaction on Predicting Subsequent Momentary Psychotic-Like and Affective Manifestations

	PRS-ES	Positive Situation Time *t*	PRS-ES × Positive Situation Time *t*	Best G × E Model^c^
Est. (S.E.)	Est. (S.E.)	Est. (S.E.)^a,b^
ESM psychosis-spectrum time *t* + 1				
ESM Paranoia				
PRS-ES (*P* < .001)	−0.015 (0.022)	−0.167 (0.024)***	−0.010 (0.018)	—
PRS-ES (*P* < .01)	0.024 (0.011)	−0.020 (0.039)***	−0.036 (0.008)***	Diathesis-stress S
PRS-ES (*P* < .05)	0.014 (0.005)	−0.037 (0.034)***	−0.019 (0.004)***	DS S
PRS-ES (*P* < .10)	0.010 (0.004)	−0.027 (0.037)***	−0.015 (0.003)***	DS S
ESM PLE				
PRS-ES (*P* < .001)	0.003 (0.012)	−0.040 (0.011)***	−0.017 (0.008)	—
PRS-ES (*P* < .01)	0.008 (0.006)	−0.034 (0.018)***	−0.005 (0.004)	—
PRS-ES (*P* < .05)	0.005 (0.003)	−0.022 (0.016)***	−0.004 (0.002)	—
PRS-ES (*P* < .10)	0.005 (0.002)	−0.035 (0.017)***	−0.002 (0.002)	—
T1 ESM negative-like symptoms				
PRS-ES (*P* < .001)	0.015 (0.048)	0.111 (0.035)*	−0.056 (0.026)	—
PRS-ES (*P* < .01)	−0.010 (0.022)	0.128 (0.058)*	−0.016 (0.012)	—
PRS-ES (*P* < .05)	0.005 (0.11)	0.034 (0.050)*	0.004 (0.006)	—
PRS-ES (*P* < .10)	0.003 (0.009)	−0.025 (0.055)*	0.009 (0.005)	—
ESM affect time *t* + 1				
ESM negative affect				
PRS-ES (*P* < .001)	0.016 (0.030)	−0.365 (0.030)***	−0.022 (0.022)	—
PRS-ES (*P* < .01)	0.014 (0.014)	−0.255 (0.049)***	−0.030 (0.010)**	Diathesis-stress W
PRS-ES (*P* < .05)	0.017 (0.007)	−0.200 (0.042)***	−0.025 (0.005)***	Diathesis-stress W
PRS-ES (*P* < .10)	0.011 (0.005)	−0.191 (0.046)***	−0.020 (0.004)***	DS W
ESM positive affect				
PRS-ES (*P* < .001)	−0.069 (0.045)	0.734 (0.049)***	0.016 (0.036)	—
PRS-ES (*P* < .01)	−0.032 (0.021)	0.551 (0.080)***	0.046 (0.016)**	Diathesis-stress W
PRS-ES (*P* < .05)	−0.027 (0.010)	0.511 (0.070)***	0.032 (0.008)***	Diathesis-stress W
PRS-ES (*P* < .10)	−0.013 (0.008)	0.479 (0.076)***	0.027 (0.007)***	DS W

*Note*: DS, differential susceptibility; ESM, experience sampling methodology; G × E, gene-by-environment interaction; PLE, psychotic-like experiences; PRS-ES, Polygenic Risk Score; S, strong; W, weak.

^a^Adjusted for ancestry PC1 and PC2.

^b^ *P* values are indicated after FDR correction for multiple testing.

^c^Complete outputs of the LEGIT competitive-confirmatory analyses are shown in Supplementary Tables 2–4.

*P < .05, **P < .01, ***P < .001.

Table 4 shows the results of the effects of stressful situations, which predicted subsequent levels of momentary paranoia, PLE, NA, and PA, but not negative-like symptoms. After adjusting for multiple testing, PRS-ES only moderated the association between the current stressful situation and subsequent paranoia (threshold *P* < .001). The interaction fitted a weak model of diathesis-stress, in this case for participants with low PRS-ES were most vulnerable to stressful situations.

**Table 4.** Effects of PRS of Environmental Sensitivity (*P* < .001, .01, .05, .10), Stressful Situation and Their Interaction on Predicting Subsequent Momentary Psychotic-Like and Affective Manifestations

	PRS-ES	Stressful Situation Time *t*	PRS-ES × Stressful Situation Time *t*	Best G × E Model
	Est. (S.E.)	Est. (S.E.)	Est. (S.E.)^a,b^	
ESM psychosis-spectrum time *t* + 1				
ESM Paranoia				
PRS-ES (*P* < .001)	−0.049 (0.024)	0.192 (0.020)***	−0.062 (0.015)***	Diathesis-stress W
PRS-ES (*P* < .01)	0.017 (0.011)	0.106 (0.033)***	0.008 (0.007)	—
PRS-ES (*P* < .05)	0.011 (0.005)	0.107 (0.028)***	0.005 (0.003)	—
PRS-ES (*P* < .10)	0.009 (0.004)	0.088 (0.030)***	0.006 (0.003)	—
ESM PLE				
PRS-ES (*P* < .001)	−0.010 (0.012)	0.064 (0.009)***	−0.015 (0.007)	—
PRS-ES (*P* < .01)	0.003 (0.006)	0.074 (0.015)***	−0.005 (0.003)	—
PRS-ES (*P* < .05)	0.005 (0.003)	0.044 (0.013)***	0.001 (0.002)	—
PRS-ES (*P* < .10)	0.005 (0.002)	0.045 (0.014)***	0.001 (0.001)	—
ESM Negative-Like Symptoms				
PRS-ES (*P* < .001)	−0.027 (0.049)	0.015 (0.029)	−0.027 (0.022)	—
PRS-ES (*P* < .01)	−0.030 (0.022)	0.067 (0.050)	−0.017 (0.011)	—
PRS-ES (*P* < .05)	0.001 (0.011)	0.056 (0.042)	−0.009 (0.005)	—
PRS-ES (*P* < .10)	0.003 (0.009)	0.042 (0.045)	−0.005 (0.004)	—
ESM affect time *t* + 1				
ESM negative affect				
PRS-ES (*P* < .001)	0.004 (0.030)	0.355 (0.024)***	−0.033 (0.019)	—
PRS-ES (*P* < .01)	0.004 (0.014)	0.355 (0.041)***	−0.006 (0.009)	—
PRS-ES (*P* < .05)	0.015 (0.006)	0.290 (0.035)***	0.005 (0.004)	—
PRS-ES (*P* < .10)	0.012 (0.005)	0.270 (0.037)***	0.006 (0.003)	—
ESM positive affect				
PRS-ES (*P* < .001)	−0.084 (0.053)	−0.478 (0.041)***	0.018 (0.032)	—
PRS-ES (*P* < .01)	−0.025 (0.025)	−0.468 (0.070)***	0.001 (0.015)	—
PRS-ES (*P* < .05)	−0.023 (0.011)	−0.469 (0.059)***	0.001 (0.007)	—
PRS-ES (*P* < .10)	−0.014 (0.010)	−0.420 (0.062)***	−0.005 (0.006)	—

*Note*: DS, differential susceptibility; ESM, experience sampling methodology; G × E, gene-by-environment interaction; PLE, psychotic-like experiences; PRS-ES, Polygenic Risk Score; S, strong, W, weak.

^a^Adjusted for ancestry PC1 and PC2.

^b^ *P* values are stated after FDR correction for multiple testing.

^c^Complete outputs of the LEGIT competitive-confirmatory analyses are shown in Supplementary table 5.

***P < .001.

## Discussion

The present study examined for the first time whether individuals with high genetic sensitivity to the environment showed differential psychotic-like and affective reactivity to positive and negative contexts in daily life. We found that PRS-ES moderated the effects of positive, but not negative, contexts on subsequent levels of paranoia, NA and PA. G × E interactions were consistent with a pattern of diathesis-stress at the lower thresholds of PRS-ES, but results supported the DS model at the highest threshold of the PRS-ES (*P* < .10). This indicates that participants with elevated scores (at more polygenic thresholds of) PRS-ES showed increased levels of momentary paranoia and NA as well as decreased PA in the subsequent assessment when reporting low positive situations, but also decreased paranoia and NA as well as increased PA when they had rated the situation as positive.

Overall, this study adds novel evidence on DS in schizotypy and PLE by showing that participants with high genetic sensitivity to environmental influences are not only differentially affected by early-life experiences (Barrantes-Vidal et al submitted in this special issue), but also by normally occurring events in daily life. Sensitivity might be related to developmental trajectories triggered by experiences that occurred in the past (ie, developmental plasticity)^28,29^ but also to the immediate effects of current environmental stimuli (ie, contextual plasticity).^28,29^ Only a few studies have focused on short-term changes when examining DS, and most of them tended to rely on experimental manipulations of the environment.^22,30–32^ For instance, at a temperamental level, Slagt and colleagues^27^ examined the moderating effect of children’s emotional reactivity, a potential susceptibility marker in infancy, on moment-to-moment interactions between parents and children.^27^ They found evidence supporting differential reactivity as highly emotional reactive children were more likely to respond with increasingly negative emotions in response to their mother’s negative emotions, but also more likely to show increasingly positive emotions in response to their mother’s positive emotions.^27^

As hypothesized, significant interactions between PRS-ES and positive context ratings were associated with subsequent levels of paranoia, NA, and PA. These interactions were consistent with a G × E model of diathesis-stress at PRS-ES threshold *P* < .01, indicating that those with high levels of PRS-ES exposed to low ratings on positive situations showed increased momentary paranoia and NA, as well as decreased PA, at the subsequent assessments. However, when the PRS-ES *P*-value threshold increased (ie, more SNPs were included into the polygenic score), interactions fitted DS. At threshold *P* < .05, the interaction between PRS-ES and positive context on paranoia was consistent with DS, and at threshold *P* < .10 all three interactions (paranoia, NA, and PA) fitted the DS model, indicating that participants with high (and more polygenic) PRS-ES were not only negatively affected by less positive situations but also showed decreased levels of paranoia and NA, as well as increased PA, when the situation was highly positive, fitting a “*for better and for worse*” pattern. Interestingly, these differential findings based on the *P-*value threshold of PRS-ES are in line with previous studies employing this polygenic score. Keers et al^53^ and Lemery-Chalfant et al^62^ also found that the thresholds *P* < .05 and *P* < .10, but not *P* < .01 or *P* < .001, of PRS-ES moderated effects of a cognitive-behavioral intervention on children’s anxiety^53^ and the effects of a family-based intervention on children’s internalizing symptoms^62^ in a “*for better and for worse*” pattern. Thus, the present study supports PRS-ES as a proxy DS factor, particularly at thresholds *P* < .05 and *P* < .10, indicating that sensitivity to the environment and (positive) daily life experiences are relevant in the mechanistic pathway to a myriad of subclinical psychopathology expressions. In line with emerging evidence about the transdiagnostic protective role of positive experiences,^16^ we found that positive context ratings predicted lower paranoia and NA, as well as greater PA—this positive impact was only found for PLE at the level of direct effects, possibly related to a reduced ability to capture interaction effects given the more limited variance of these experiences in our non-clinical sample. This finding is particularly relevant for the psychotic dimension, as research has traditionally focused on the detrimental effects of adversity, with scant attention to the potential impact of positive environmental factors. Furthermore, this may have important clinical implications for the design of ecological momentary interventions from a positive psychology perspective, aimed at promoting resilience-building and well-being rather than only focusing on diminishing risk for symptom expression and maintenance.^68^

As expected, and in line with previous evidence,^26^ PRS-ES did not moderate the effects of positive context ratings on negative psychotic-like symptoms. In contrast, we did expect that PRS-ES moderated the effects of positive context on subsequent PLE, as we found a DS pattern when examining the PRS-ES moderation of childhood adversity on positive PLE retrospectively measured.^26^ It is relevant to highlight that the assessment of PLEs can be challenging, especially in daily life and in non-clinically ascertained college-student samples.^69^ As compared to paranoia, one of the most prevalent psychotic manifestations reported in the general population (with prevalence 10%)^70,71^ that can be ascertained by perceptions of suspiciousness and mistrust, PLEs are a diverse and heterogeneous phenomenon that can include unusual perceptual experiences (visual, olfactory, auditory, etc.) and odd beliefs. This may partly explain their low endorsement rates and variability, which impacts the power of detecting interaction effects.

Contrary to our hypothesis, the PRS-ES did not yield interactions with stressful contexts, except for one interaction with the lowest threshold of PRS-ES (*P* < .001) predicting paranoia. However, given the lack of consistency across thresholds and the fact that it was an isolated effect compared to the multiple consistent effects of PRS-ES on the association between positive context and subclinical phenomena, this finding should be considered cautiously. Our ability to capture significant interaction effects might have been limited by the fact that the item “*My current situation is stressful*” showed a lower endorsement (*M* = 2.15, SD = 1.06) than “*My current situation is positive"* (*M* = 5.35, SD = 0.96). From the total number of assessments (*n* = 7526), more than half (ie, 57.1%) were endorsed as “1” (“Not at all”), indicating the absence of stress. Nevertheless, we did find main effects of stress on subsequent levels of momentary paranoia, PLE, NA, and PA, consistent with previous evidence on psychotic and affective stress-reactivity in daily life across the psychosis continuum.^36,38–43^ Of note, in another study using the same sample, we showed that a PRS indexing variability of the hypothalamic–pituitary–adrenal (HPA) axis function, one of the main neural systems involved in regulating the stress response, moderated the effects of momentary stressful appraisals on subsequent levels of NA in daily life (Torrecilla et al unpublished data). Thus, it might be speculated that the PRS-ES may not be a powerful moderator of the impact of mild daily stressors as other genetic indicators more directly indexing biologically functional variability in stress-regulating systems.

The present study has several strengths and limitations. In order to overcome replicability limitations of candidate-gene research, we employed a polygenic approach based on a twin-based GWAS predicting pair-differences in internalizing problems.^52^ Thus, in contrast to traditional GWAS designs, characterized by aggregating small genetic main effects, the PRS-ES is a combination of variants associated with the magnitude of twins’ response to their non-shared environments. Nonetheless, given the complex genetic architecture of environmental sensitivity and the multidimensionality underlying the trait, as shown by Assary et al,^72^ twin differences in emotional problems may not capture the full picture of genetic influences underlying sensitivity to environment. Further research may refine the measurement of environmental sensitivity to fully disentangle the genetic influences involved in sensitivity to both, positive and negative exposures. The use of ecologically valid measures of real-life context and subclinical experiences across multiple time points over a week in real life is a notable strength of the present study. Specifically, the examination of ESM time-lagged associations between contextual appraisals and subsequent psychological states enables to estimate causal inferences of the effects of the predictor on the criterion. Altogether, the use of real-time measurement in combination with polygenic approaches has been suggested to greatly advance our understanding of G × E in psychopathology and well-being as well as enhancing G × E reliability research.^51^ Finally, the study of G × E factors using a non-clinical sample with a wide distribution of schizotypy traits is useful to understand the etiology of full-blown manifestations (ie, schizophrenia) without the confounding effects associated to clinical status, such us pharmacology treatment or hospitalization.^4,5^

Findings of the present study should be interpreted with caution due to several limitations. First, the use of a high-quality intensive repeated assessment method over a week (ESM) conditioned a reduction of sample size. However, momentary assessment technologies have been suggested to substantially reduce sample size requirements and substantially enhance the detection of subtle G × E effects.^73^ Furthermore, the use of a predominantly female university student sample limits generalizability.

The present findings offer a promising initial examination of DS in schizotypy, but ultimately require replication in larger independent samples with more representative distributions of gender, age, and educational levels. Results are in line with the notion that environmental sensitivity may be a crucial transdiagnostic causative factor of diverse psychopathology dimensions and highlight the potential value of protective factors such as minor daily positive experiences. Further support to this research would stress the value of positive intervention and prevention strategies focused on decreasing poor mental health outcomes and increasing well-being in highly sensitive individuals.

## Supplementary Material

Supplementary material is available at https://academic.oup.com/schizophreniabulletin/.

sbad162_suppl_Supplementary_Tables_1-5
