# Supplementary material for: Genetic Differences in Reactivity to the Environment Impact Psychotic-Like and Affective Reactivity in Daily Life
Source: Schizophr Bull. 2025 Mar 4;51(Suppl 2):S74–84. doi: 10.1093/schbul/sbad162 (PMC11879516; doi:10.1093/schbul/sbad162)
Supplement: sbad162_suppl_Supplementary_Tables_1-5 [file sbad162_suppl_supplementary_tables_1-5.docx]

**Supplementary Materials**

*Genotyping, Quality Control and Imputation*

DNA was extracted from saliva or cotton swabs samples using the following extraction kits: i) the prepIT-L2P kit (DNA Genotek Inc., Ottawa, Ontario, Canada) for saliva samples and ii) the RealPure Genomic DNA Extraction Kit (Durviz S.L.U., Valencia, Spain) for cotton swab samples. DNA samples were genotyped at the “Centro Nacional de Genotipado” (CEGEN-PRB3-ISCIII; CNIO-Madrid) using the Illumina Infinium Global Screening Array-24 v2.0 (GSA) BeadChip. Genotype calls were generated with GenomeStudio v2.0.4 (Illumina Inc., San Diego, CA, USA). The quality control (QC) was performed using PLINK v1.9 (www.cog-genomics.org/plink/1.9/)^1^. During QC, SNPs were excluded when: had a missing call rate >2%; had a Minor Allele Frequency (MAF) <0.1%; or deviated from Hardy-Weinberg equilibrium with a P-value <0.001. Subjects were excluded when they had a missing call rate >2%; were genetically related to other participants or duplicated samples according to the pairwise identity by descent method (PI_HAT >0.25); or had non-European ancestry according to a Multidimensional Scaling (MDS) analysis, which was carried out with PLINK v1.9 to obtain a representation of genetic ancestry in our study, extracting the first 10 ancestry components. From the total sample at Time 2 of 253 non-clinical individuals, 25 subjects were excluded during QC leaving a sample of 228 subjects. MDS components were recalculated in this final sample and the two first components were used in all models including PRS as independent variables. Imputation was performed using the Haplotype Reference Consortium panel (www.haplotype-reference-consortium.org)^2^ in the Michigan Imputation Server^3^. A post-imputation QC was carried out to exclude SNPs that had an imputation quality score of R2 <0.3; or had a MAF <1%. A total of 7,755,414 SNPs passed post-imputation QC.

**References**

1. Chang CC, Chow CC, Tellier LC, Vattikuti S, Purcell SM, Lee JJ. Second-generation PLINK: rising to the challenge of larger and richer datasets. *Gigascience*. 2015;4:7.
2. McCarthy S, Das S, Kretzschmar W, et al. A reference panel of 64,976 haplotypes for genotype imputation. *Nat Genet*. 2016;48(10):1279-1283.
3. Das S, Forer L, Schönherr S, et al. Next-generation genotype imputation service and methods. *Nat Genet*. 2016;48(10):1284-1287

**Table 1. Bootstrapped Pearson correlations for the study variables (n=217).**

|  |  | **1** | **2** | **3** | **4** | **5** | **6** | **7** | **8** | **9** | **10** | **11** |
| --- | --- | --- | --- | --- | --- | --- | --- | --- | --- | --- | --- | --- |
| **1. PRS-ES (*p*<.001)** | r | - | .39*** | .40*** | .37*** | -.00 | -.14* | -.04 | -.00 | -.01 | .05 | -.15* |
|  | 95% CI^a^ |  | [.28, .51] | [.28, .51] | [24, .49] | [-.14, .13] | [-.25, -.02] | [-.19, .13] | [-.17, .18] | -.14, .12] | [-.09, .18] | [-.28, -.03] |
| **2. PRS-ES (*p*<.01)** | r |  | - | .67*** | .61*** | -.02 | -.10 | .07 | .09 | -.05 | .04 | -.05 |
|  | 95% CI |  |  | [.58, .73] | [.52, .68] | [-.16, .12] | [-.23, .03] | [-.05, .22] | [.00, .20] | [-.15, .06] | [-.10, .17] | [-.19, .08] |
| **3. PRS-ES (*p*<.05)** | r |  |  | - | .88*** | .03 | -.18** | .12 | .11 | .07 | .11 | -.14* |
|  | 95% CI |  |  |  | [.85, .91] | [-.10, .18] | [-.31, -.06] | [.00, .24] | [.00, .26] | [-.04, .17] | [-.01, .24] | [-.26, -.02] |
| **4. PRS-ES (*p*<.10)** | r |  |  |  | - | -.02 | -.17* | .10 | .15* | .08 | .08 | -.08 |
|  | 95% CI |  |  |  |  | [-.15, .12] | [-.31, -.04] | [-.01, .23] | [.07, .27] | [-.03, .17] | [-.03, .20] | [-.20, .05] |
| **5. ESM Stressful situation** | r |  |  |  |  | - | -.39*** | .36*** | .39*** | .01 | .62*** | -.53*** |
|  | 95% CI |  |  |  |  |  | [-.53, -.23] | [.23, .49] | [.27, .56] | [-.09, .12] | [.51, .71] | [-.63, -.42] |
| **6. ESM Positive situation** | r |  |  |  |  |  | - | -.36*** | -.29*** | -.10 | -.53*** | .81*** |
|  | 95% CI |  |  |  |  |  |  | [-.48, -.23] | [-.46, -.16] | [-.24, .03] | [-.62, .43] | [.75, .86] |
| **7. ESM Paranoia** | r |  |  |  |  |  |  | - | .70*** | .18*** | .76*** | -.34*** |
|  | 95% CI |  |  |  |  |  |  |  | [.60, .80] | [.02, .39] | [.67, .83] | [-.46, -.22] |
| **8. ESM PLE** | r |  |  |  |  |  |  |  | - | .24*** | .58*** | -.28*** |
|  | 95% CI |  |  |  |  |  |  |  |  | [.07, .39] | [.48, .74] | [-.47, -.15] |
| **9. ESM Negative symptoms** | r |  |  |  |  |  |  |  |  | - | .09 | -.01 |
|  | 95% CI |  |  |  |  |  |  |  |  |  | [-.07, .30] | [-.16, .14] |
| **10. ESM Negative Affect** | r |  |  |  |  |  |  |  |  |  | - | -.56*** |
|  | 95% CI |  |  |  |  |  |  |  |  |  |  | [-.64, -.48] |
| **11. ESM Positive Affect** | r |  |  |  |  |  |  |  |  |  |  | - |
|  | 95% CI |  |  |  |  |  |  |  |  |  |  |  |

⁎p < 0.05. ⁎⁎ p < 0.01. ⁎⁎⁎ p < 0.001. a Bootstrapped 95% confidence intervals for N=1000 samples.

PRS-ES=Polygenic Risk Score of Environmental Sensitivity. ESM=Experience Sampling Methodology.

**Table 2. LEGIT competitive-confirmatory tests for significant interaction between PRS-ES and positive situation on paranoia.**

| *Outcome:*  *ESM Paranoia time t + 1* | | PRS-ES (p=.01) x ESM Positive situation | | PRS-ES (p=.05) x ESM Positive situation | | PRS-ES (p=.10) x ESM Positive situation | |
| --- | --- | --- | --- | --- | --- | --- | --- |
|  |  | AIC | Crossover point (95%) | AIC | Crossover point (95%) | AIC | Crossover point (95%) |
| GxE  models | DS STRONG | 8865.91 | 0.65 ( 0.57 / 0.74 ) | **8864.45** | **0.67 ( 0.59 / 0.75 )** | **8866.64** | **0.60 ( 0.51 / 0.68 )** |
|  | DS WEAK | 8872.31 | 0.67 ( 0.58 / 0.75 ) | 8870.23 | 0.71 ( 0.63 / 0.8 ) | 8872.9 | 0.62 ( 0.54 / 0.7 ) |
|  | Diathesis STRONG | **8865.82** | **1** | 8864.77 | 1 | 8867.55 | 1 |
|  | Diathesis WEAK | 8872.31 | 1 | 8869.45 | 1 | 8872.74 | 1 |
|  | Vantage STRONG | 8896.76 | -1 | 8906.68 | -1 | 8900.56 | -1 |
|  | Vantage WEAK | 8886.55 | -1 | 8888.52 | -1 | 8888.42 | -1 |
| Non-GxE models | Intercept only | 16037.17 | NA | 16037.17 | NA | 16037.17 | NA |
|  | G only | 16029.17 | NA | 16004.19 | NA | 16012.18 | NA |
|  | E only | 10145.17 | NA | 10145.17 | NA | 10145.17 | NA |
|  | G+E only | 10145.92 | NA | 10143.38 | NA | 10144.7 | NA |

*Note: Best model indicated by lowest AIC is* ***highlighted****.*

**Table 3. LEGIT competitive-confirmatory tests for significant interaction between PRS-ES and positive situation on negative affect.**

| *Outcome:*  *ESM Negative affect at time t + 1* | | PRS-ES (p=.01) x ESM Positive situation | | PRS-ES (p=.05) x ESM Positive situation | | PRS-ES (p=.10) x ESM Positive situation | |
| --- | --- | --- | --- | --- | --- | --- | --- |
|  |  | AIC | Crossover point (95%) | AIC | Crossover point (95%) | AIC | Crossover point (95%) |
| GxE  models | DS STRONG | 11338.57 | 0.48 ( 0.43 / 0.54 ) | 11314.84 | 0.57 ( 0.52 / 0.62 ) | 11314.45 | 0.48 ( 0.43 / 0.53 ) |
|  | DS WEAK | 11316.48 | 0.48 ( 0.43 / 0.53 ) | 11298.97 | 0.67 ( 0.62 / 0.72 ) | **11303.5** | **0.55 ( 0.5 / 0.6 )** |
|  | Diathesis STRONG | 11344.94 | 1 | 11321.11 | 1 | 11322.68 | 1 |
|  | Diathesis WEAK | **11315.85** | **1** | **11298.61** | **1** | 11304.02 | 1 |
|  | Vantage STRONG | 11389.5 | -1 | 11395.21 | -1 | 11379.25 | -1 |
|  | Vantage WEAK | 11320.46 | -1 | 11318.29 | -1 | 11318.28 | -1 |
| Non-GxE models | Intercept only | 20686.08 | NA | 20686.08 | NA | 20686.08 | NA |
|  | G only | 20685.03 | NA | 20645.63 | NA | 20665.48 | NA |
|  | E only | 12763.54 | NA | 12763.54 | NA | 12763.54 | NA |
|  | G+E only | 12765.13 | NA | 12762.2 | NA | 12764.95 | NA |

*Note: Best model indicated by lowest AIC is* ***highlighted****.*

**Table 4. LEGIT competitive-confirmatory tests for significant interaction between PRS-ES and positive situation on positive affect.**

| *Outcome:*  *ESM Positive affect at time t + 1* | | PRS-ES (p=.01) x ESM Positive situation | | PRS-ES (p=.05) x ESM Positive situation | | PRS-ES (p=.10) x ESM Positive situation | |
| --- | --- | --- | --- | --- | --- | --- | --- |
|  |  | AIC | Crossover point (95%) | AIC | Crossover point (95%) | AIC | Crossover point (95%) |
| GxE  models | DS STRONG | 17292.41 | 0.54 ( 0.49 / 0.58 ) | 17289.7 | 0.61 ( 0.56 / 0.66 ) | 17278.35 | 0.43 ( 0.38 / 0.47 ) |
|  | DS WEAK | 17251.12 | 0.65 ( 0.61 / 0.7 ) | 17242.7 | 0.85 ( 0.81 / 0.89 ) | **17244.61** | **0.47 ( 0.43 / 0.51 )** |
|  | Diathesis STRONG | 17300.07 | 1 | 17296.76 | 1 | 17293.15 | 1 |
|  | Diathesis WEAK | **17249.84** | **1** | **17241** | **1** | 17245.44 | 1 |
|  | Vantage STRONG | 17366.84 | -1 | 17392.23 | -1 | 17356.68 | -1 |
|  | Vantage WEAK | 17256.08 | -1 | 17257.6 | -1 | 17254.36 | -1 |
| Non-GxE models | Intercept only | 29593.37 | NA | 29593.37 | NA | 29593.37 | NA |
|  | G only | 29592.05 | NA | 29537.16 | NA | 29580.66 | NA |
|  | E only | 18237.1 | NA | 18237.1 | NA | 18237.1 | NA |
|  | G+E only | 18238.78 | NA | 18232.89 | NA | 18237.36 | NA |

*Note: Best model indicated by lowest AIC is* ***highlighted****.*

**Table 5. LEGIT competitive-confirmatory tests for significant interaction between PRS-ES and stressful situation on psychotic-like experiences (PLE).**

| *Outcome:*  *ESM PLE at time t + 1* | | PRS-ES (p=.01) x ESM Stressful situation | |
| --- | --- | --- | --- |
|  |  | AIC | Crossover point (95%) |
| GxE  models | DS STRONG | -127.54 | -0.47 ( -0.89 / -0.05 ) |
|  | DS WEAK | -167.27 | -0.75 ( -0.91 / -0.59 ) |
|  | Diathesis STRONG | -129 | -1 |
|  | Diathesis WEAK | **-169.13** | **-1** |
|  | Vantage STRONG | -126.51 | 1 |
|  | Vantage WEAK | -166.04 | 1 |
| Non-GxE models | Intercept only | 4001.27 | NA |
|  | G only | 4002.38 | NA |
|  | E only | 1968.37 | NA |
|  | G+E only | 1968.78 | NA |

*Note: Best model indicated by lowest AIC is* ***highlighted****.*
